# Spatial Metagenomics of Three Geothermal Sites in Pisciarelli Hot Spring Focusing on the Biochemical Resources of the Microbial Consortia

**DOI:** 10.3390/molecules25174023

**Published:** 2020-09-03

**Authors:** Roberta Iacono, Beatrice Cobucci-Ponzano, Federica De Lise, Nicola Curci, Luisa Maurelli, Marco Moracci, Andrea Strazzulli

**Affiliations:** 1 Department of Biology, University of Naples “Federico II”, Complesso Universitario Di Monte S. Angelo, Via Cupa Nuova Cinthia 21, 80126 Naples, Italy; roberta.iacono@unina.it (R.I.); nicola.curci@unina.it (N.C.); 2 Institute of Biosciences and BioResources, National Research Council of Italy, Via P. Castellino 111, 80131 Naples, Italy; beatrice.cobucciponzano@ibbr.cnr.it (B.C.-P.); federica.delise@ibbr.cnr.it (F.D.L.); luisa.maurelli@ibbr.cnr.it (L.M.); 3 Task Force on Microbiome Studies, University of Naples Federico II, 80134 Naples, Italy

**Keywords:** origin of life, microbial community, CAZymes, extremozymes, environmental changes, comparative metagenomics

## Abstract

Terrestrial hot springs are of great interest to the general public and to scientists alike due to their unique and extreme conditions. These have been sought out by geochemists, astrobiologists, and microbiologists around the globe who are interested in their chemical properties, which provide a strong selective pressure on local microorganisms. Drivers of microbial community composition in these springs include temperature, pH, in-situ chemistry, and biogeography. Microbes in these communities have evolved strategies to thrive in these conditions by converting hot spring chemicals and organic matter into cellular energy. Following our previous metagenomic analysis of Pisciarelli hot springs (Naples, Italy), we report here the comparative metagenomic study of three novel sites, formed in Pisciarelli as result of recent geothermal activity. This study adds comprehensive information about phylogenetic diversity within Pisciarelli hot springs by peeking into possible mechanisms of adaptation to biogeochemical cycles, and high applicative potential of the entire set of genes involved in the carbohydrate metabolism in this environment (CAZome). This site is an excellent model for the study of biodiversity on Earth and biosignature identification, and for the study of the origin and limits of life.

## 1. Introduction

Extreme environments such as hot springs are of great interest as a source of novel extremophilic microorganisms, enzymes, and metabolic pathways essential for the microbial survival in extreme conditions [1]. Extremophiles are known to thrive in diverse extreme conditions, such as high or low temperatures, high salinity, acidic and alkaline pH values, and high radiation [2]. They not only can tolerate these conditions but require the latter for survival. Exploring the diversity of extremophiles and understanding their mechanisms of adaptation [3] permit us to expand our notions of the potential habitable environments able to sustain life beyond Earth [4]. Indeed, sites harboring harsh environments identified during solar system exploration may host now, or may have hosted, extremophilic life forms.

Research on extremophiles and their enzymes (extremozymes) has not only reshaped our understanding of the origin and evolution of life [5] and the potential for life on other planetary bodies [6], but also it has simultaneously led to numerous advances in molecular biology, medicine, and biotechnology [7,8,9,10]. In fact, extremozymes represent interesting cases of protein adaptation under conditions where conventional enzymes quickly denature [11,12,13]. Thus, extremozymes are ideal tools for industrial applications where harsh chemical and physical conditions are encountered. However, the difficulties in cultivating extremophiles severely limit access to this class of biocatalysts, thereby metagenomic approaches are now largely used for extremozyme discovery [14,15,16,17].

The increasing amounts of metagenomic data and fully sequenced genomes now allow us to systematically explore these microbial communities [14,18], enabling us to investigate the uncultured microbial population, the mechanisms of possible adaptation to biogeochemical cycles, and the lifestyles of extreme organisms, and to discover new extremozymes [19].

Recently, our group reported on the metagenomic analysis of the microbial community populating the Pisciarelli hot springs (Naples, Italy), identifying the entire carbohydrate active enzymes portfolio (CAZome) that has been cloned and partially characterized [20].

In detail, two main mud/water pools have been identified in Pisciarelli in March 2012, named Pool 1 (T = 85 °C, pH 5.5) and Pool 2 (T = 92 °C, pH 1.5). The first pool was almost exclusively populated by Archaea (*Acidianus hospitalis,* 40%; *Pyrobaculum arsenaticum,* 20%; *Pyrobaculum oguniense,* 5%; *Saccharolobus solfataricus,* 1%) followed by Bacteria (0.11%) and archaeal viruses (0.17%), while more than 30% of the obtained reads found no match with the nucleotide NCBI database. In contrast, the majority of the obtained reads from Pool 2 found no match with the NT NCBI database (62%). Among the assigned reads, Crenarchaeota (37%) dominate the site (*Metallosphaera sedula,* 31%; *Saccharolobus solfataricus,* 3%; *Acidianus hospitalis*, 3%) followed by archaeal viruses (0.36%) [20]. In that study, we demonstrated that even sites that have been consistently sampled for decades are still largely unexplored in terms of microbial diversity and of their extremozymes. The microbial population in Pisciarelli has been shown to have a huge number of genes encoding putative CAZymes, which include glycoside hydrolases (GHs), carbohydrate esterases (CEs), polysaccharide lyases (PLs), and auxiliary activities (AAs) [20]. These activities are classified in the CAZy database (www.cazy.org) [21]. Thus, these biocatalysts are ideal candidates for biotechnological applications and to understand enzyme adaptations to extreme environments.

In the last 15 years, the geothermal activity in the Pisciarelli area has been increasing, showing a rise in fumarolic discharge, the formation of boiling pools and water springs (March 2009), and the opening of energetic geyser-type vents (November 2010) that are currently very active. The temperature at the main Pisciarelli fumarole rose up to 110 °C in 2011, but dropped sharply in April 2012 to the present value of 95 °C [22,23]. In December 2012, the Italian Civil Protection Department raised the alert of the caldera from green level (base) to the current yellow level (attention) as a consequence of a further increase in the deformation rate, seismicity, and degassing [22]. Currently, the hydrothermal activity at Pisciarelli shows an escalation characterized by an increase in the CO_2_ flux, which in 2019 exceeded 500 t/day [24].

For these reasons, we embarked on a novel metagenomic study of the Pisciarelli hot springs in order to explore the microbial communities populating mud/water pools formed as a result of the local changes that occurred.

To date, Pisciarelli represents a unique ecological niche for comparative metagenomics studies. The access to this site provides valuable insight into the adaptive strategies of the extremophiles communities, and conditions generally difficult to study in other remote extreme environments and/or to reproduce in lab. The study can lead to a more comprehensive understanding of the mechanisms of evolutionary change that underlie the adaptation of microbes to extreme conditions.

## 2. Results and Discussion

### 2.1. Sampling in the Solfatara Hot Springs, mDNA Extraction, and Sequencing

**Figure 1:**
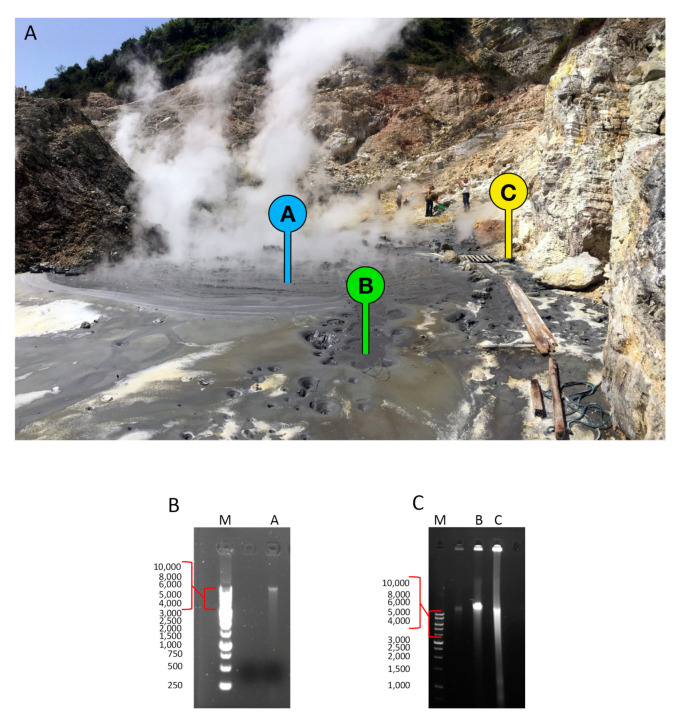
(**A**) View of the sampling site in July 2019. The sites A, B, and C are labeled by badges. (**B**) Agarose gel mDNA extraction from Site A. Lane M, Marker StoS 1Kb DNA Ladder (Genespin), lane A: mDNA from Site A. (**C**) Agarose gel of mDNA extractions from sites B and C. Lane M, Marker StoS 1Kb DNA Ladder (Genespin); lane B, mDNA from Site B; lane C, mDNA from Site C.

The aim of this study is to explore the microbial communities populating three sites of the Pisciarelli hot springs (40°49′45.1″ N; 14°8′49.4″ E), named Site A, Site B, and Site C (Figure 1A) and investigate their differences in terms biodiversity and potential source of enzymes.

Site A (94.1 °C; pH 5.2) is the largest pool of the area, mainly made by water and mud. Recently, the microbial community populating Site A (then called Pool 1) has been characterized through a metagenomic approach showing the dominance of Archaea belonging to the genera *Acidianus* (40%) and *Pyrobaculum* (25%), as well as the presence of sequences related to new phyla ascribable to the Sulfolobaceae family. A dramatic morphological change in the area of interest, which occurred at the end of July 2019, led to the expansion of the area previously known as Pool 1, which merged to the proximal Pool 2 pool (92 °C, pH 1.5), generating a completely new site (Site A). This new pool, if compared to Pool 1, changed both in terms of extension of the pool boundaries and in terms of temperature, showing, in particular, an increase of about 9 °C compared to the conditions previously observed [20].

Site B (47.7 °C; pH 5.8) is mainly composed of mud and is physically adjacent to Site A from which it receives part of the liquid fraction. Nonetheless, Site B has its emission of gas, which can be observed through the formation of bubbles that contribute to the mixing of the liquid on the surface.

Site C (73.0 °C; pH 2.5) differently, is physically distant from Site A and Site B and is a shallow pool of water located near to the rocky wall of the area (Figure 1A) and characterized by intense steam jets.

Temperature and pH were monitored in-situ at the three sites and the samples, composed of water and sediment, were collected and taken to the laboratory where, by centrifugation, sediments of 17 g, 50 g, and 40 g were obtained from Sites A, B, and C, respectively.

The sediment obtained from each sample was treated for the extraction of the whole mDNA obtaining 27, 60, and 164 ng/g of sediment from Site A, Site B, and Site C, respectively (Figure 1B,C).

The mDNA was then sequenced in outsourcing by Novogene-Europe (Cambridge, UK) through Illumina MiSeq (150 PE), obtaining 23,830,104 clean reads from Site A, 22,933,864 from Site B, and 23,961,446 from Site C.

### 2.2. Microbial Communities

To evaluate the composition of the microbial communities populating the three sites, the obtained reads were analyzed by *blastn* against the NCBI NT nucleotide database.

**Figure 2:**
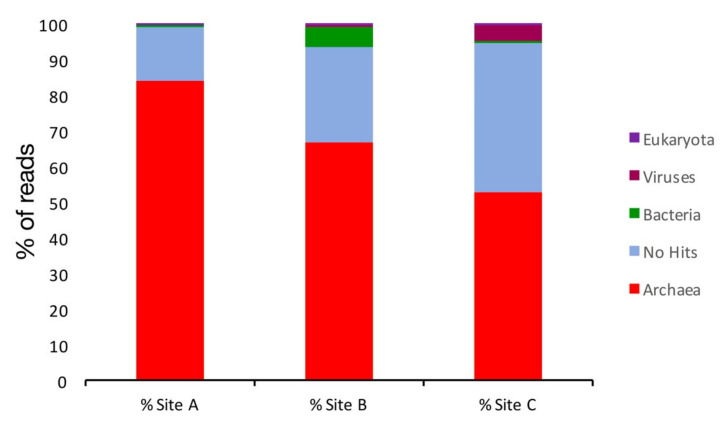
Taxonomic assignment of the reads at the kingdom level.

The analysis revealed that all three sites are dominated by Archaea, and Site A had the highest number of reads (84%) assigned to this kingdom, followed by Site B (67%) and Site C (53%) (Figure 2). It is worth noting that each site showed a high number of reads that had no match in the NT database (unassigned). Notably, unassigned reads represented 15, 27, and 41% of the whole reads of Site A, B, and C, respectively. Furthermore, Site B had the highest number of reads assigned to the kingdom of Bacteria (5%) compared to the Sites A and C, where less than 1% of sequences could be assigned to this kingdom. Site C, on the contrary, showed a percentage of viral sequences (4.6%) higher than Sites A and B (0.3 and 0.8%, respectively).

**Figure 3:**
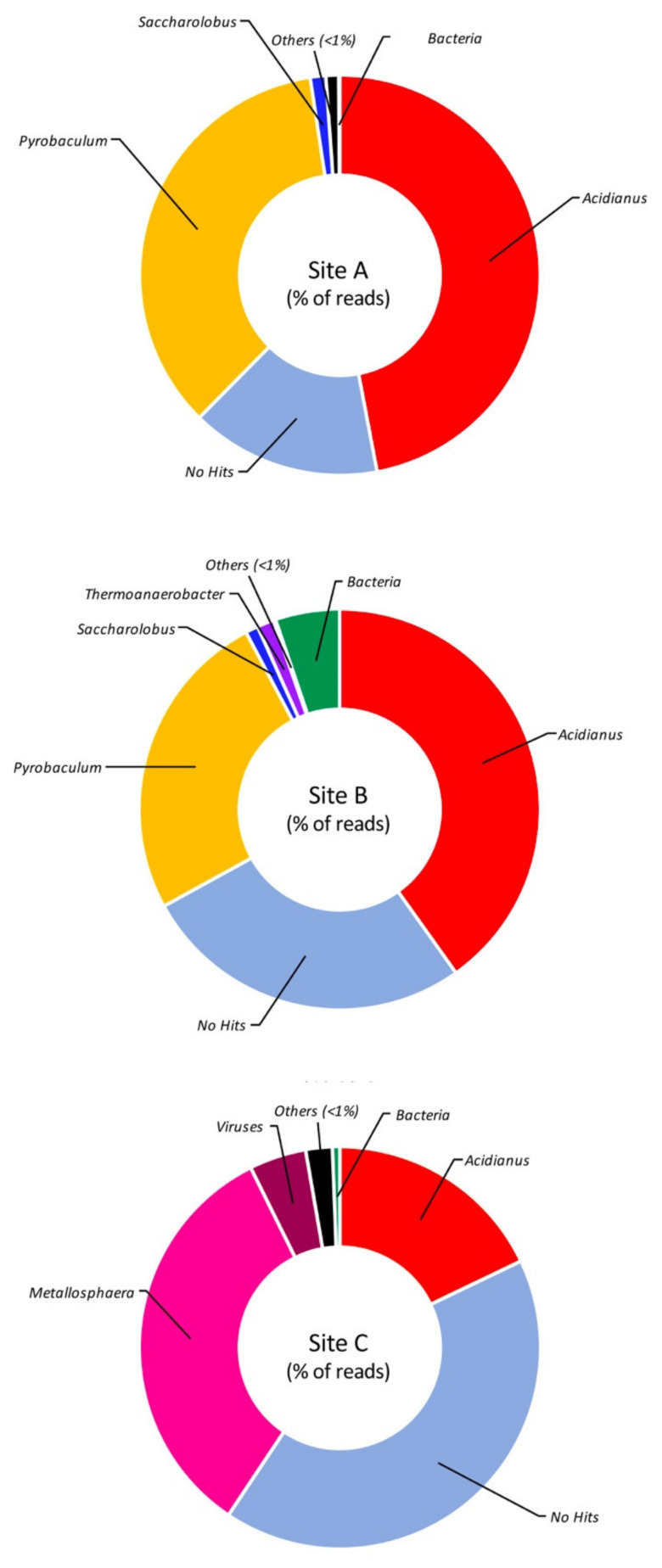
Taxonomic assignment of the reads at the genus level. Taxa showing less than 1% of assigned reads are grouped as “others”.

**Figure 4:**
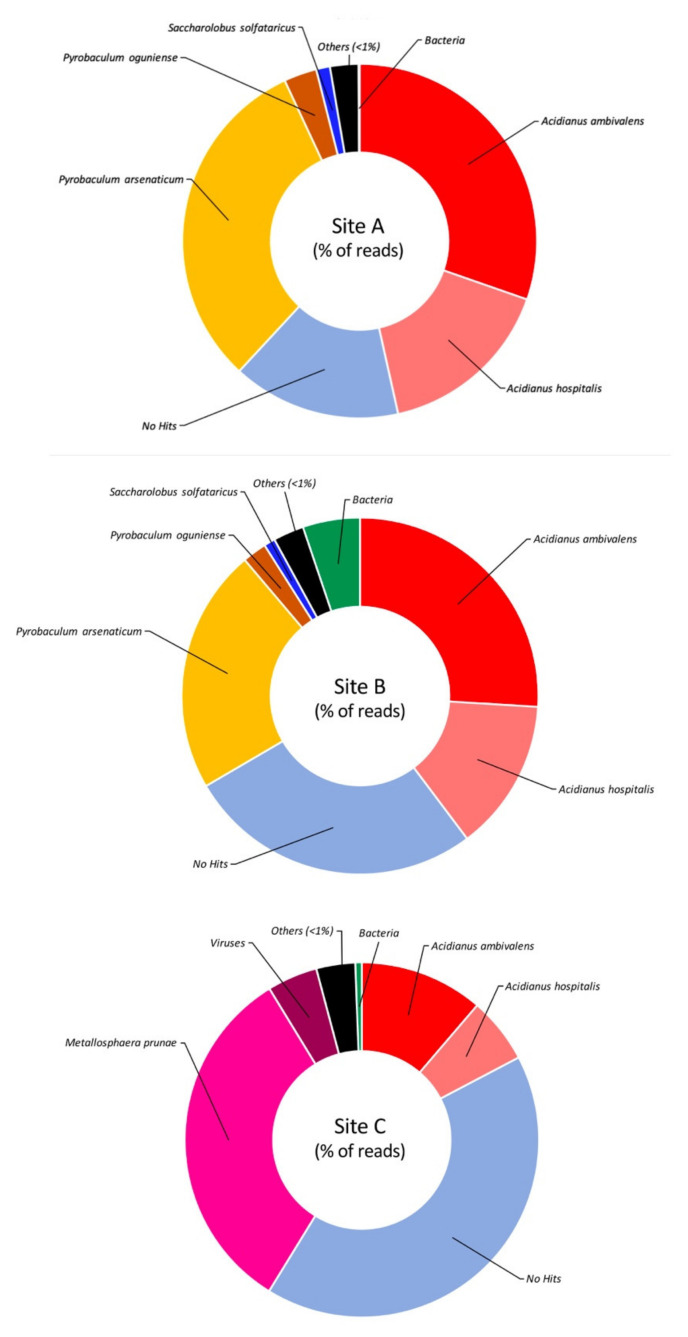
Taxonomic assignment of the reads at the species level. Taxa showing less than 1% of assigned reads are grouped as “others”.

The detailed investigation of the taxonomically assigned reads, at the level of genus and species (Figure 3 and Figure 4), revealed that Site A is mainly dominated by the genus *Acidianus* (47%), in particular the species *A. ambivalens* (30%) and *A. hospitalis* (16%), followed by the genus *Pyrobaculum* (35%) mainly attributable to the species *P. arsenaticum* (31%). This result differs from what was previously observed on the Pool 1 site in which we observed the dominance of *A. hospitalis* (40%) followed by *P. arsenaticum* (20%) [20].

A notable difference between the result observed in Site A and the previous study consists in the percentage of reads of unknown origin, which decreased from 32% in Pool 1 to 15.4% in Site A. This variation indicates a change in biodiversity of the microbial community populating the pool as an effect of the geothermal events that occurred between March 2012 and July 2019 (Pool 1 Biosample ID: SAMN09692669).

Site B, alike Site A, also showed *Acidianus* (40%) as the dominant genus, followed by *Pyrobaculum* (25%) (Figure 3) with a relative abundance of species comparable to what was observed in Site A (Figure 4). This parallelism can be explained considering that the two sites are not physically distant to each other and that the liquid fraction present in Site B was partially provided by the proximal Site A (Figure 1A). Indeed, the main difference between Site A and Site B is that in the latter, 5% of the reads were assigned to bacterial origins and the number of sequences not assigned to known microorganisms was 1.7-fold higher than those present in Site A.

Reads assigned to the genus *Acidianus* were also present in the sample from Site C. These, however, represented only 18% of the total reads, while the dominant genus was represented by *Metallosphaera* (33%) (Figure 3), mainly attributable to the species *M. prunae*, which is known to grow at temperatures between 55 and 80 °C and pH range 1.0–4.5 [25]. This result is in line with what was previously observed in another extremely acidic site of the Pisciarelli hot spring, named Pool 2 (92 °C, pH 1.5) (Pool 2 Biosample ID: SAMN09692670), where *Metallosphaera sedula* was the dominant species [20]. Nonetheless, the most abundant component (41.5%) of Site C consisted of reads that did not match any known sequence.

Another remarkable difference between Site C and the other two sites was the number of viral reads identified in this sample. As mentioned above, 4.6% of Site C reads were taxonomically assigned to viruses. Among these, viruses mainly belonging to Bicaudaviridae (different variants of *Acidianus* two-tailed virus and disparate types of *Sulfolobus monocaudavirus*), Ampullaviridae (*Acidianus* bottle-shaped viruses), Fuselloviridae (*Sulfolobus* spindle-shaped viruses), and Ligamenvirales (*Sulfolobales* rod-shaped viruses) were identified.

It is known that viruses play a key role in horizontal gene transfer (HGT) in prokaryotes [26]. Transfer of DNA has been shown to be involved in genome evolution and in adaptation to high temperatures [27]. In particular, it has been proven that spindle-shaped fuselloviruses that infect *Sulfolobus* and *Acidianus* species can promote the virus-mediated HGT between different hosts [28], contributing significantly to the dynamic of the prokaryotic genomes. Thus, the presence in high percentages of viral sequences in Site C might be attributed as a survival mechanism against rapid environmental changing of this extreme site. Therefore, the peculiar microbial composition of Site C is presumably related to the considerably more acidic pH value if compared to the other two mud pools.

### 2.3. Analysis of Bacteria Communities

To evaluate the bacterial communities present in Sites A, B, and C, the reads of the three samples were analyzed in detail in the NT database (Table 1).

**Figure 5:**
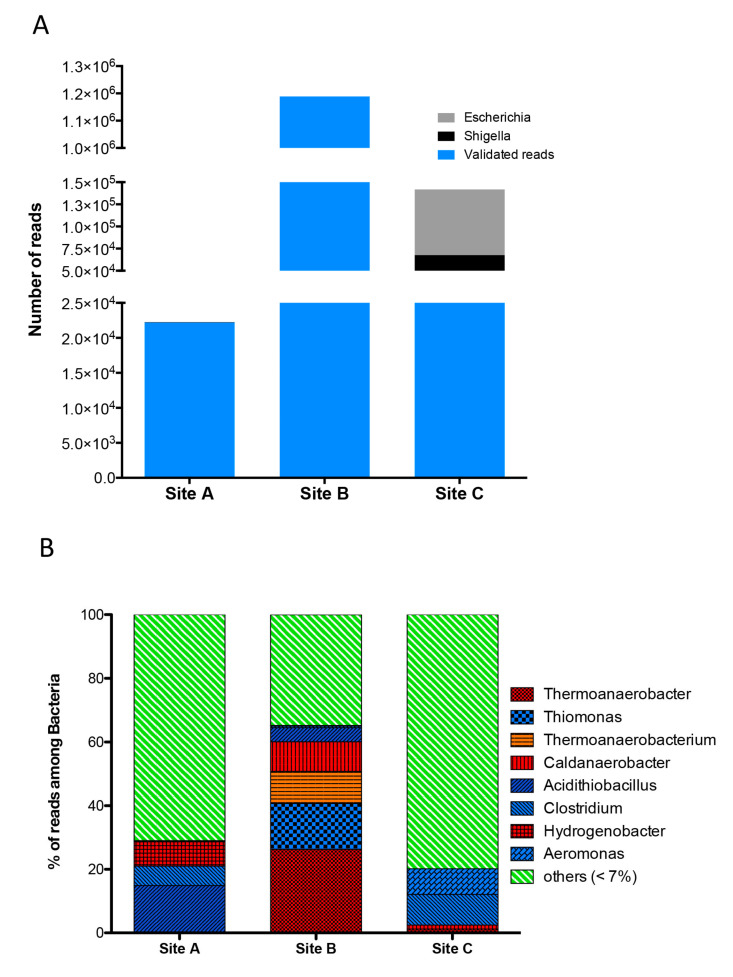
(**A**) Number of reads in Sites A, B, and C assigned to bacteria. Black and grey indicate the reads assigned to the genera *Shigella* and *Escherichia*, respectively, and filtered as contaminants. In blue the number of validated reads used for the bacteria community analysis. (**B**) Bacteria community profile, relative abundances, and diversity: hyperthermophiles (red), thermophiles (orange), mesophiles (blue), others (each relative abundance % < 7, green).

In Site A, 22,276 were assigned to bacteria (Figure 5A) whose most abundant genera were represented by the mesophilic/moderately thermophilic bacterium *Acidithiobacillus* (15%) and the hyperthermophilic *Hydrogenobacter* (8%), while the remaining 78% belonged to different genera whose relative abundance was less than 7% (Figure 5B). Regarding the genus *Acidithiobacillus* it is important to highlight that, although this is mainly represented by mesophilic microorganisms, it also groups the moderately thermophilic *Acidithiobacillus caldus* with an optimal growth pH between 2.0–2.5 and with an optimal temperature of 45 °C [32].

As previously indicated, among the three sites, Site B showed by far the highest percentage of bacterial reads (1,188,674). The analysis of the relative abundances of bacteria present in the microbial community of Site B (Figure 5B) allowed us to identify the hyperthermophiles *Thermoanaerobacter* (26%) and *Caldanaerobacter* (9%), the thermophile *Thermoanaerobacterium* (10%), and *Acidithiobacillus*, which, unlike in Site A, was much less abundant here (4%). It is important to note that one of the most abundant bacterial genus present in Site B was *Thiomonas* (14%), which, although generally grouping mesophilic species, also includes moderately thermophilic species identified in geothermal sources at ~45 °C and able to grow at temperatures up to 50 °C and in the pH range 4.0–7.0 [29,30,31].

In Site C, 141,784 reads were assigned to the kingdom of Bacteria. Of these, 15 and 52% were assigned to the genera *Shigella* and *Escherichia*, respectively (Figure 5A). Since both are mesophilic gammaproteobacteria whose natural habitat is the human and animal gut [33], these reads were considered as environmental contaminations and not taken into account for the purpose of evaluating the bacterial population.

Among the remaining 46,141 reads of Site C, the most abundant genera were *Clostridium* (10%) and *Aeromonas* (8%). The *Clostridium* genus includes obligate anaerobic bacteria and Gram-positive bacteria and are capable of forming spores in adverse environmental conditions, which populate soil, sand, rivers, swimming pools, river bank mud, and marine sediments [34]. Unfortunately, the low number of reads from Site C, associated with this genus, has not allowed for a more detailed taxonomic annotation, and it is therefore currently impossible to trace the species present in the sample. However, recently, two studies on microbial communities populating Malaysian hot springs (temperatures range 50–110 °C) [35] and five hot springs in Eritrea (temperatures between 45 and 100 °C) [36] revealed the presence of various human pathogens, including *Clostridium spp*. and *Aeromonas*. In addition, a novel thermophilic *Clostridium* species (*C. thermarum*) from a thermal spring in China has recently been identified and characterized [37].

### 2.4. Assembly, Clustering, and Taxonomic Analysis of Unassigned Reads

To identify possible chunks of individual genomes present in the three samples, all the reads were separately assembled by MEGAHIT [38] obtaining 6296, 38,136, and 16,854 contigs in Sites A, B, and C, respectively (Table 2).

Contigs with a length ≥1000 bp were analyzed by MyCC [39], thus allowing the identification of 25 clusters in Site A, 21 clusters in Site B, and 16 clusters in Site C (Figure S1).

The clusters obtained were then analyzed by CheckM [40], which allowed us to validate those with completeness values ≥20%, obtaining five clusters in Site A (7, 12, 17, 22, and 23), fifteen clusters in Site B (2, 3, 4, 6, 7, 8, 9, 11, 13, 14, 15, 16, 18, 19, and 20), and five clusters in Site C (1, 4, 5, 8, and 10) (Tables S1 and S2).

To obtain a taxonomic assignment, the validated clusters were analyzed by Diamond (in blastx mode) using the NCBI Refseq Protein database [41] (Table 3). The result of this analysis made it possible to note the Site A clusters as belonging to the *Crenarchaeota* phylum, in particular related to the genera *Acidianus*, *Pyrobaculum*, and *Desulfurococcus*.

Site C, on the contrary, was characterized by clusters entirely related to *Acidianus spp*. and by two clusters (4 and 5) with a highly heterogeneous assignment that prevented the identification of a dominant species.

Differently, the clusters identified in Site B were mainly assigned to bacteria belonging to the phyla Firmicutes and Proteobacteria, confirming what was already observed from the taxonomic analysis of the reads. Three of the clusters of Site B were assigned to the *Crenarchaeota* phylum, related to *Desulforococcus* spp. and *P. arsenaticum.*

To identify the origin of the reads that had no match against the NCBI NT nucleotide database, these reads were aligned using Bowite2 [42] (Table 4). Regarding the unassigned Site A reads, most of them were aligned with clusters 9, 22, and 23. The first two clusters were assigned to the genus *Acidianus* (Table S1) and presumably represented the result of HGT, as previously reported [20].

Instead, cluster 23 was assigned to the genus *Hydrogenobacter*, suggesting the possible presence of microorganisms not yet identified belonging to the Aquificaceae family.

As regards the unassigned Site B reads, these were aligned mainly against clusters 7 and 13. While cluster 13 was assigned to *Pyrobaculum arsenaticum,* indicating also in this case a probable HGT event, cluster 7 was instead taxonomically identified only at the family level as Sulfolobaceae.

The unassigned reads of Site C represented a completely special case. Indeed, these mostly aligned (>85%) to cluster 10, whose taxonomic analysis was classified as related to the genus *Acidianus*. Observing the contamination value of cluster 10 (45%) (Table S1), with which CheckM indicated the percentage of the expected number of duplicate single-copy markers, it was legitimate to assume that this cluster had grouped contigs belonging to species of *Acidianus* not yet identified.

### 2.5. Evaluation of Microbial Replication Rates

To evaluate the individual contribution to the metabolic functions of the microbial consortia present in Sites A, B, and C, the replication indices of the validated clusters were calculated using iRep [43].

Among all the analyzed clusters, only ten respected the selection parameters (Table S3) and were analyzed with iRep, which was able to determine the replication index for only four of these: cluster 7 of Site A and clusters 2, 4, and 6 of Site B (Table 5).

The obtained replication indices allowed us to estimate the percentage of replication of the microbial species associated with the clusters indicating that in Site A and in Site B, *Desulfurococcus*, despite the low number of reads assigned to this taxon (<0.1% of the total reads in both sites), had more than 30% of the cells in active replication (Figure S2).

In addition, regarding Site B, iRep showed that the bacteria belonging to the order of Thermoanaerobacterales (2.4% of the total reads), including *Caldanaerobius*, had more than 50% of the cells in the duplication phase (Figure S2).

Unfortunately, it was not possible to calculate the replication index of the other clusters, probably due to the limitation of iRep, which during the analysis discarded the regions with very high and very low coverage, applying a linear regression model relating exclusively to the coverage of the region containing the origin of replication [43].

### 2.6. Functional Annotation and CAZome Analysis

**Figure 6:**
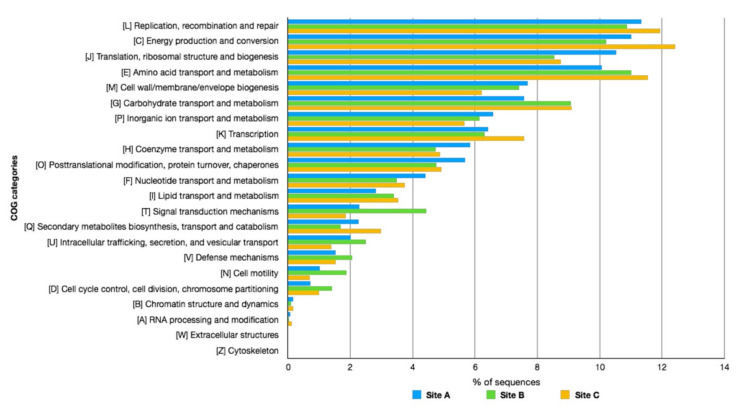
COG analysis of the metagenomes in Pisciarelli hot springs. Sites A, B, and C are compared according to COG functional categories.

**Figure 7:**
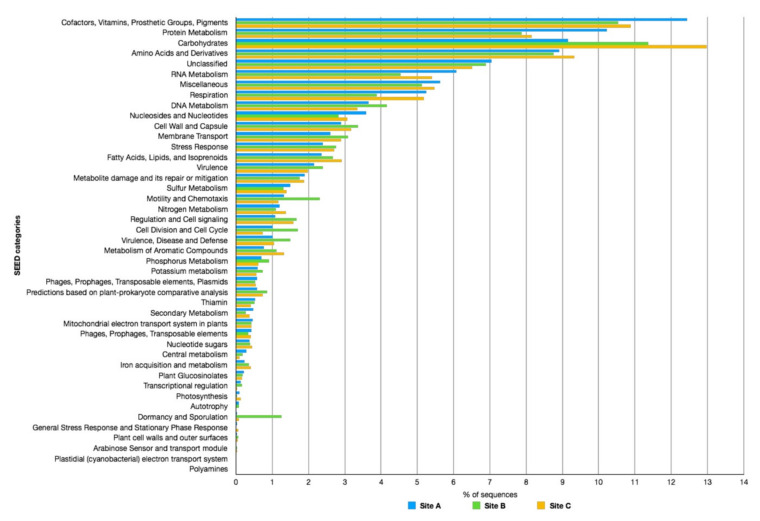
Functional annotation according to the SEED database of the metagenomes in Pisciarelli sites A, B, and C.

To assess the metabolic potential of the microbial consortia populating the three sites, the contigs obtained by the assembly were analyzed by Prodigal [44] identifying 14,933 ORFs on Site A, 81,938 ORFs on Site B, and 31,179 ORFs on Site C. Then, the amino acid sequences of the identified ORFs were functionally classified using the COG and SEED databases (Figure 6 and Figure 7).

The analysis of the three samples showed an average comparable distribution of the functional categories reported in both databases. However, by observing in more detail the classification according to the COG database (Figure 6), it is possible to observe a marked difference in relation to the sequences assigned to the functional category “Signal transduction mechanisms” (Category T), where the percentage of ORFs of Site B was two-fold greater than those of Sites A and C. A more in-depth analysis of the ORFs assigned to this category revealed that this difference was mainly due to the high number of ORF annotated histidine kinases of bacterial origin.

In addition, the annotation using the SEED database (Figure 7) showed an average homogeneous distribution between the functional classes, but there were clear differences in relation to the category “Protein Metabolism”, more abundant in Site A; “Carbohydrates”, more abundant in Site C, where it was also the most represented category; as well as in the categories “Mobility and Chemotaxis” and “Dormancy and Sporulation”, in which Site B clearly dominated the other two. In particular, regarding the “Dormancy and Sporulation” category, this was mainly composed of ORFs annotated as Stages 0, I, II, III, IV, and V sporulation proteins, confirming the presence of different sporogenic bacteria in this site.

As for the “Mobility and Chemotaxis” category, the main differences were related to the presence of ORFs of bacterial origin involved in the structure and mobility of the flagellum, while in Site C, the ORFs annotated as “Archaeal Flagellum” and “Bacterial Chemotaxis” (dipeptide-binding ABC transporter) were most abundant.

In all three samples, the largest number of ORFs functionally annotated belonged to the categories “Carbohydrate Transport and Metabolism” and “Carbohydrate” of COG and SEED, respectively. A similar result was previously observed in Pool 1 and Pool 2 and related to the abundant vegetation around the Pisciarelli thermal spring, rich in starch, hemicellulose, and pectins, which could represent an available carbon source for the microbial communities populating these geothermal sites [20].

To map the difference of the enzymatic activities involved in the synthesis, degradation, and modification of carbohydrates (CAZymes) in the Sites A, B, and C, the ORFs of the three samples were analyzed by dbCAN2 [45]. The assessment of the taxonomic origin of the identified CAZymes revealed that in Sites A and C, the highest number of CAZymes belonged to the phylum of the Crenarchaeota (76 and 93% respectively), while 71% of the CAZymes identified in Site B belonged to the phylum of the Firmicutes (Table 6).

**Figure 8:**
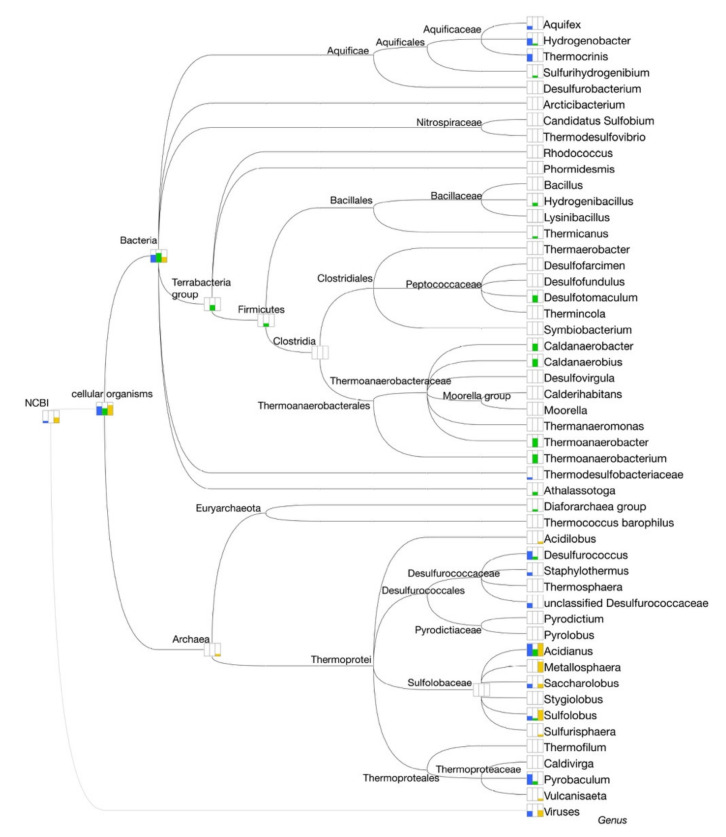
Taxonomic assignment of the CAZymes identified in sites A, B, and C. The filled area of the rectangle indicates, on a logarithmic scale, the number of ORFs assigned to each taxon.

The genus analysis (Figure 8) indicated a higher number of *Acidianus*-related CAZymes for Sites A and C. In addition, while Site A had numerous activities related to *Pyrobaculum* and *Desulfurococcus* and to several (hyper)thermophilic bacteria of the phylum Aquificae (*Hydrogenobacter*, *Thermocrinis*, and *Aquifex*), the CAZymes of Site C were mainly assigned to the genera *Metallosphaera, Saccharolobus*, and *Sulfolobus*. Differently, the greater number of CAZymes identified in Site B belonged to the thermophilic bacteria of the genus *Thermoanaerobacterium*, *Thermoanaerobacter*, *Caldanaerobius*, *Caldanaerobacter*, and *Desulfotomaculum* (Figure 8).

**Figure 9:**
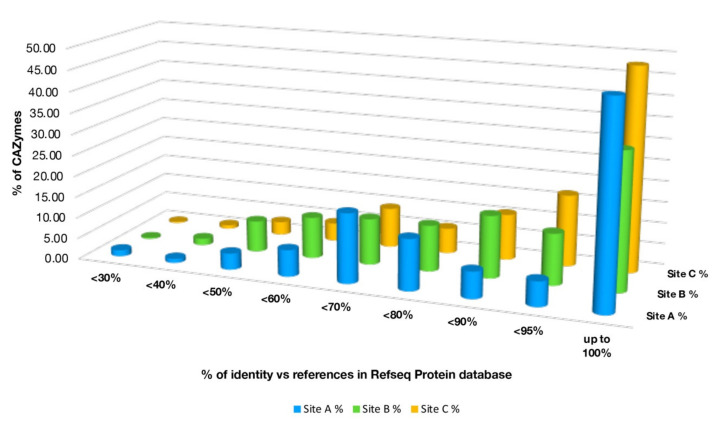
Identity percentages of the CAZymes annotated in the sites A, B, and C against homologs in the Refseq Protein Database.

However, although it was possible to annotate the identified CAZymes at the genus level, less than 50% of these had an identity ≥95% compared to sequences already present in the Refseq Protein Database, indicating the presence of new sequences related to carbohydrate active enzymes (Figure 9, Tables S4–S6).

**Figure 10:**
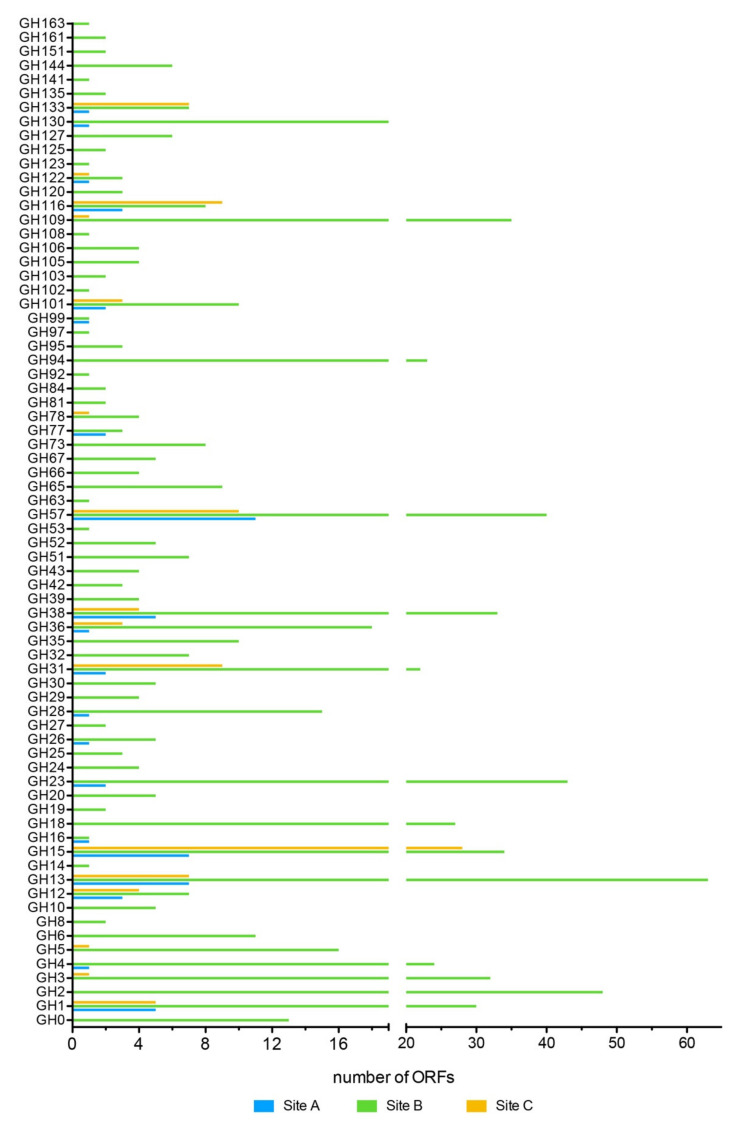
Distribution of glycosidases among the sites A, B, and C. The ORF number assigned to GHs from each sample is displayed.

Glycosidases (GHs) represented 28%, 43%, and 27% of the CAZymes identified in Site A, Site B, and Site C, respectively. Among the families of GHs common in all three sites (Figure 10, Table 7), GH13, GH15, GH31, GH57, GH122, and GH133 were identified, which groups enzymes mainly active on α-glycosidic bonds, indicating the presence of pathways of degradation of starch and amylopectins used as energy reserves in plants around the area (mainly ferns, dicots, and grass) [20].

Among the identified GHs, 48 of these were present only in Site B (Table 7). The analysis of aminoacidic sequences showed that these were associated exclusively with (hyper)thermophilic bacteria with identities between 33% and 100% compared to the sequences present in the NCBI NR database (Table S5).

A large fraction of the identified CAZymes was annotated as glycosyltransferase (GTs). In particular, in Sites A and C they represented the most abundant activity class (62% and 58% of the CAZymes, respectively), while in Site B GTs were only 38%.

**Figure 11:**
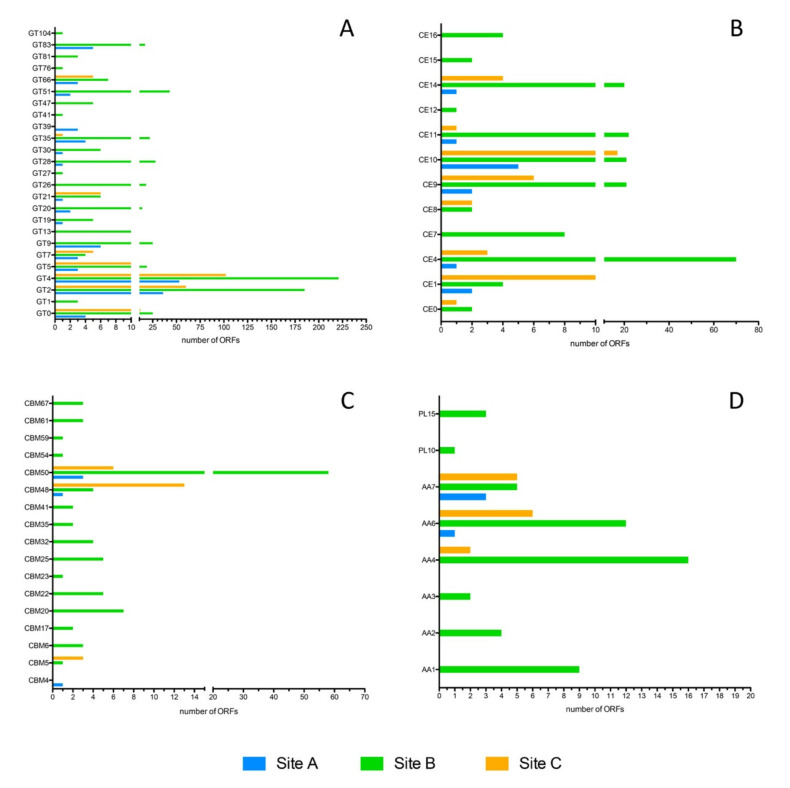
Distribution of the (**A**) glycosyltransferase, (**B**) carbohydrate esterases, (**C**) carbohydrate-binding modules, (**D**) auxiliary activities and polysaccharide lyases among the Pisciarelli sites. The ORFs number assigned to each family from the three samples is displayed.

Eight hypothetical families of GTs were identified exclusively in Site B (Figure 11A), namely GT1, GT13, GT27, GT41, GT76, GT81, and GT104, which are involved in glycosylation mechanisms of proteins and peptides, and GT47, which groups heparan β-glucuronyltransferase, xyloglucan β-galactosyltransferase, heparan synthase, and arabinan α-L-arabinosyltransferase. The aminoacidic sequence of the ORFs classified in these families revealed identity percentages between 48% and 99% compared to the sequences present in NR database, and a bacterial origin related to the genera *Acidithiobacillus*, *Anaeromusa*, *Caldanaerobacter*, *Desulfofarcimen*, *Desulfotomaculum*, *Desulfurella*, *Planifilum*, *Syntrophorhabdus*, *Thermoanaerobacter*, *Thermoanaerobacterium*, and *Thiomonas*.

In addition, with regard to carbohydrate esterases (CEs) (Figure 11B), Site B showed unique families (CE7, CE12, CE15, and CE16) which group putative acetyl xylan esterases, pectin acetyl esterases, rhamnogalacturonan acetyl esterases, 4-O-methyl-glucuronoyl methylesterases, and acetyl-mannan esterases with identity percentages between 58 and 100% with CEs identified in members of the genera *Thermoanaerobacter* and *Desulforella* (Table S5), and which might be involved in the metabolism of hemicellulose polysaccharides.

Site B has also been shown to be particularly rich in hypothetical carbohydrate-binding modules (CBMs), in auxiliary activities (AAs) and in polysaccharide lyases (PLs) (Figure 11C,D). The hypothetical CBMs identified exclusively in Site B are mainly involved in the degradation of starch and amylopectin (CBM20, CBM25, CBM41) and of cellulosic and hemicellulosic polysaccharides (CBM6, CBM22, CBM23, CBM32, CBM54, CBM59) with an identity percentage between 43% and 99% with bacterial sequences, mostly associated with the *Thermoanaerobacter* and *Caldanaerobacter* genera (Table S5). In contrast, the only exclusive CMB family identified in Site A was CBM4, which groups specific modules for xylan, β-1,3-glucan, β-1,3-1,4-glucan, β-1,6-glucan, and amorphous cellulose but not crystalline cellulose. The single sequence in Site A, annotated as CBM4, shows 97% identity with the cellulase C (GH16) from *Cellvibrio mixus* containing a CBM4 [46].

The hypothetical AAs present in all three sites belong to the families AA6 (1,4-benzoquinone reductases) and AA7 glucooligosaccharide oxidases and chitooligosaccharide oxidases, showing >80% identity in Sites A and C with sequences mainly associated with the phylum of the Crenarchaeota. In Site B, the sequences of which more than 50% of identity have mainly been attributed to the bacterial phyla of Firmicutes and Proteobacteria as well as for the families AA1, AA2, and AA3, were identified exclusively in this sample.

Finally, only in Site B ORFs annotated as PLs were found. Among these, in particular, there was a sequence assigned to the PL10 family, which includes pectate lyase, and three sequences assigned to the PL15 family, which includes alginate lyase, oligoalginate lyase/exo-alginate lyase, heparin lyase I, and heparin lyase III. While the only identified PL10 showed a low identity (47%) with the pectate lyase from *Pelosinus* sp. *UFO1*, the three ORFs classified as PL15 showed identities between 90 and 99% with hypothetical proteins associated with the thermophilic genera *Thermoanaerobacter* and *Caldanaerobacter* (Table S5).

The remarkable number of sequences encoding putative CAZymes makes the Pisciarelli microbial population an attractive source of novel thermophilic biocatalysts for industrial applications. The functional annotation here described represents a preliminary survey, but already promising a relevant biochemical potential of the microbial consortia of the different geothermal sites in the Pisciarelli area. Indeed, future research will carry out more detailed studies on these extremophilic communities and their CAZomes in order to deepen their knowledge and exploit their biodiversity in biotechnological processes.

## 3. Materials and Methods

### 3.1. Pisciarelli Hot Springs Sampling

Samples from the hydrothermal mud/water pools, Site A, Site B, and Site C, respectively, were transferred into sterile tubes, respectively, were closed and immediately transferred to the laboratory for DNA extraction. In situ measurements of temperature and pH were performed by using an HI-93510 thermometer (HANNA instruments, Padova, Italy) equipped with a Pt100 probe, and a pH meter for field use (sensION^TM^ + PH1 equipped with 5051T electrode (HACH)).

### 3.2. Isolation of DNA

The samples composed by mud/water were centrifuged at 6000× *g* for 20 min at RT, and the sediments were stored at −20 °C. The metagenomic DNA samples were purified from 5 g of sediment collected from each site by following the protocol previously reported [47], except for the lysis step performed by freeze–thawing in dry ice and at 65 °C.

The amounts of obtained metagenomic DNA were quantified by a Qubit 4 Fluorometer using the Qubit™ DNA HS assay kit (Invitrogen-Thermo Fisher Scientific corporation, Waltham, Massachusetts, USA), and DNA quality was independently assessed by visualization on 1% agarose (*w*/*v*) gels.

### 3.3. Sequencing of mDNA

The extracted and purified mDNA of each site was used for shotgun sequencing with Miseq (Illumina) performed at Novogene Europe, Cambridge (UK), and the datasets obtained were provided as clean reads. The sequencing reads are available in the NCBI Sequence Read Archive (SRA) database under the accession numbers SRR12124857 (Site A), SRR12124856 (Site B), and SRR12124855 (Site C).

The environmental data relative to the Sites A, B, and C (NCBI BioProject PRJNA643424) are available in the Biosamples database under the accession numbers SAMN15414048 (Site A), SAMN15414049 (Site B), and SAMN15414050 (Site C).

### 3.4. Taxonomic Analysis and Assembly

For microbial diversity analysis, short paired-end Illumina reads (150 bp) were aligned to the nucleotide reference database of NCBI NT by using Blast+/BlastN. The resulting output data of each sample (Site A: reads = 21,090,866 with match in NT; Site B: reads: 18,258,802 with match in NT; Site C: reads: 15′458′279) were used as input for MEGAN6 Community Edition with the following parameters: MinScore = 40.0, MaxExpected = 0.7, TopPercent = 10.0, MinSupportPercent = 0, mode = BlastN) [48].

Clean reads were assembled using MEGAHIT [38] by using min-count = 2 and k-mers 21, 31, 41, 51, 61, 71, 81, 91, and 99.

Obtained contigs ≥1000 bp were grouped into bins by using MyCC [39]; the obtained clusters were validated by CheckM v1.0.12 [40], and reads of each sample were aligned by using Bowtie 2 [42].

### 3.5. Replicative Estimation

To obtain a replicative estimation, the clusters validated by CheckM were filtered by using an ad hoc pipeline to remove contigs with shorter than 5000 bp, completeness <75%, contamination >2%, and a ratio fragment/Mbp > 175. Again, the reads of each sample were aligned to the remaining clusters, and the SAM files obtained were analyzed by iREP [43].

### 3.6. Functional Annotation

All the contigs obtained by the assembly procedure were analyzed by using Prodigal [44] to identify the open reading frames. ORFs were analyzed by using Diamond in blastp mode [41] against the NR database and functionally classified by MEGAN6 regarding the SEED and COG databases (MinScore = 35; MaxExpected = 0.01; Top percent = 10; Min support percent = 0.05).

To identify the hypothetical carbohydrate-active enzymes the ORFs were analyzed by the dbCAN2 pipeline [45], and the resulting reads were taxonomically assigned using Diamond in bastp mode, against the NCBI Refseq Protein database.

## 4. Conclusions

Extremophiles are organisms capable of adapt themselves, survive and thrive in hostile habitats that were previously thought to be adverse or lethal for life [49]. Extreme conditions drive the evolution of their inhabitants, highlighting the role of extremophiles as models for the study of the origin and evolution of life on Earth and provide key insights into the boundaries of life, allowing us to speculate mightily about possible extraterrestrial life forms [50]. Furthermore, the molecular and physiological properties and the remarkable adaptive capabilities of extremophiles make them an attractive source of biocatalysts for diverse applications in biotechnology, biomedicine, and industrial processes.

However, the study of extremophiles is a rather difficult field, mainly constrained by the complexity of reaching their ecological niches and isolating these microbes. Pisciarelli Solfatara hot springs represents a unique ecological niche for the study of hyperthermophiles. This area, so surprisingly dynamic, is affected by sudden geothermal changes such as the increase of the magmatic component of fumaroles, frequent seismic swarms, and bradyseism, indicating that the hydrothermal system undergoes repeated injections of magmatic fluid [22]. These sudden changes are generating hostile environments for survival and growth of (hyper)thermophilic microbial life forms.

The comparative metagenomic study reported here allowed us to understand the complexity of the microbial community in three new sites that were generated by geochemical change happening in this area in July 2019. This study demonstrates that these sites, although very close to each other, showed remarkable differences in terms of pH and temperature that were reflected by significant differences in the microbial consortia inhabiting each site.

Living at such selective pressure might foster the development and retention of a suite of metabolic and physiological adaptations, which could play a key role in ensuring the presence and persistence of life in extreme environments [51].

Indeed, the metagenomic investigation revealed a broad CAZome, correlated to the abundant vegetation present around the Pisciarelli thermal spring, rich in starch, hemicellulose, and pectins, which represent a considerable carbon source for the microorganisms populating the geothermal sites.

The presence of highly sophisticated mechanisms of adaptation together with the availability of specific biochemical pathways sustaining peculiar physiological metabolic capabilities makes the extremophilic microbial communities of Pisciarelli interesting from an astrobiological point of view.

## Figures and Tables

**Table 1:** Relative abundances of the bacteria communities in the Sites A, B, and C.

Genus	Site A (%)	Site B (%)	Site C (%)	Temperature Range
*Thermoanaerobacter*	0.10	26.00	1.24	Hyperthermophilic
*Thiomonas*	0.10	14.00	0.00	Mesophilic/Moderately thermophilic ^a^
*Thermoanaerobacterium*	0.00	10.00	0.00	Thermophilic
*Caldanaerobacter*	0.00	9.00	1.24	Hyperthermophilic
*Acidithiobacillus*	15.00	4.00	0.00	Mesophilic/Moderately thermophilic ^b^
*Clostridium*	6.00	0.50	10.00	Mesophilic
*Hydrogenobacter*	8.00	0.10	0.00	Hyperthermophilic
*Aeromonas*	0.10	0.10	8.00	Mesophilic
others (< 7% of bacterial reads)	70.70	36.30	79.52	mixed

^a^ [29,30,31]. ^b^ [32].

**Table 2:** Assembly statistics.

	Site A	Site B	Site C
**Number of contigs**	6296	38,136	16,854
**Total contigs length**	9,459,744	53,411,732	18,962,457
**Mean length**	1502	1400	1125
**SD value**	160	33	54
**Max contigs length**	485,160	482,809	417,005
**Min contigs length**	200	200	200
**N50 value**	24,403	3592	2299
**N80 value**	737	727	602
**N90 value**	414	461	382

**Table 3:** Taxonomic assignment of Sites A, B, and C CheckM validated clusters.

Sample	Cluster	Phylum	Dominant Taxa
Site A	Cluster 7	Crenarchaeota	*Desulfurococcus*
	Cluster 12	Crenarchaeota	*Pyrobaculum arsenaticum*
	Cluster 17	Crenarchaeota	*Pyrobaculum* spp.
	Cluster 22	Crenarchaeota	*Acidianus hospitalis*
	Cluster 23	Crenarchaeota	*Acidianus* spp.
Site B	Cluster 2	Mixed	mixed
	Cluster 3	Proteobacteria	*Acidithiobacillus caldus*
	Cluster 4	Firmicutes	*Caldanaerobius* spp.
	Cluster 6	Crenarchaeota	*Desulfurococcus* spp.
	Cluster 7	Crenarchaeota	*Sulfolobaceae*
	Cluster 8	Firmicutes	*Thermoanaerobacterium* spp.
	Cluster 9	Firmicutes	*Thermoanaerobacter* spp.
	Cluster 11	Proteobacteria	*Thiomonas* spp.
	Cluster 13	Crenarchaeota	*Pyrobaculum arsenaticum*
	Cluster 14	Firmicutes	*Caldanaerobacter subterraneus*
	Cluster 15	Firmicutes	*Hydrogenibacillus* spp.; *Thermicanus* spp.
	Cluster 16	Firmicutes	*Desulfotomaculum copahuensis*
	Cluster 18	Proteobacteria	*Desulfurella* spp.
	Cluster 19	Firmicutes	*Caldanaerobius* spp.
	Cluster 20	Mixed	mixed
Site C	Cluster 1	Crenarchaeota	*Acidianus brierleyi*
	Cluster 4	Mixed	mixed
	Cluster 5	Mixed	mixed
	Cluster 8	Crenarchaeota	*Acidianus* spp.
	Cluster 10	Crenarchaeota	*Acidianus* spp.

**Table 4:** Percent of reads without match vs. NT aligned to each cluster.

Site A		Site B		Site C	
**Cluster**	% of Unassigned Reads Aligned	Cluster	% of Unassigned Reads Aligned	Cluster	% of Unassigned Reads Aligned
Cluster 1	0.59	Cluster 1	0.15	Cluster 1 *	0.51
Cluster 2	2.98	Cluster 2 *	3.71	Cluster 2	0.32
Cluster 3	0.05	Cluster 3 *	0.77	Cluster 3	0.1
Cluster 4	5.02	Cluster 4 *	3.83	Cluster 4 *	0.45
Cluster 5	1.59	Cluster 5	0.36	Cluster 5 *	2.17
Cluster 6	1.07	Cluster 6 *	3.48	Cluster 6	1.58
Cluster 7*	11.9	Cluster 7 *	36.51	Cluster 7	0.48
Cluster 8	0.05	Cluster 8 *	0.43	Cluster 8 *	4.4
Cluster 9	13.93	Cluster 9 *	2.43	Cluster 9	0.07
Cluster 10	1.04	Cluster 10	1.84	Cluster 10 *	85.64
Cluster 11	1.22	Cluster 11 *	8.85	Cluster 11	0.05
Cluster 12 *	4.17	Cluster 12	0.08	Cluster 12	0.68
Cluster 13	8.38	Cluster 13 *	12.92	Cluster 13	0.29
Cluster 14	0.17	Cluster 14 *	0.38	Cluster 14	0.61
Cluster 15	3.71	Cluster 15 *	8.65	Cluster 15	2.43
Cluster 16	0.22	Cluster 16 *	2.62	Cluster 16	0.22
Cluster 17 *	2.79	Cluster 17	2.04		
Cluster 18	5.73	Cluster 18 *	6.75		
Cluster 19	1.23	Cluster 19 *	1.88		
Cluster 20	0.05	Cluster 20 *	2.19		
Cluster 21	0.76	Cluster 21	0.13		
Cluster 22 *	14.89				
Cluster 23 *	18.2				
Cluster 24	0.02				
Cluster 25	0.24				

* Clusters validated by CheckM.

**Table 5:** iRep indices for validate clusters in sites A, B, and C.

Sample	Cluster	iRep Index	Dominant Taxa
Site A	Cluster 7	1.32	*Desulfurococcus*
Site B	Cluster 11	n/a	*Thiomonas* spp.
	Cluster 13	n/a	*Pyrobaculum arsenaticum*
	Cluster 15	n/a	*Hydrogenibacillus* spp.; *Thermicanus* spp.
	Cluster 18	n/a	*Desulfurella* spp.
	Cluster 2	1.57	Thermoanaerobacteriales
	Cluster 4	1.51	*Caldanaerobius* spp.
	Cluster 6	1.39	*Desulfurococcus* spp.
	Cluster 7	n/a	*Sulfolobaceae*
	Cluster 9	n/a	*Thermoanaerobacter* spp.
Site C	Cluster 5	n/a	mixed
	Cluster 10	n/a	*Acidianus* spp.

n/a: not applicable.

**Table 6:** Taxonomic assignment of CAZymes.

Phylum	Site A	Site B	Site C
Crenarchaeota	76.0	15.2	92.8
Aquificae	16.4	2.9	0
Viruses	3.8	0.6	5.6
Thermodesulfobacteria	1.3	0.2	0
Euryarchaeota	0.6	2.2	0.4
Nitrospirae	0.0	1.7	0
Thermotogae	0.0	2.9	0
Firmicutes	0.0	71.1	0.8
Others (<1%)	1.9	3.2	0.4

**Table 7:** Shared GHs families among the sites.

Sites	Number of GHs Families	Shared GHs Families
Site A, Site B, and Site C	12	GH1, GH12, GH13, GH15, GH31, GH36, GH38, GH57, GH101, GH116, GH122, GH133
Site A and Site B	8	GH4, GH16, GH23, GH26, GH2, GH77, GH99, GH130
Site B and Site C	4	GH3, GH5, GH78, GH109
Site B	48	GH0, GH2, GH6, GH8, GH10, GH14, GH18, GH19, GH20, GH24, GH25, GH27, GH29, GH30, GH32, GH35, GH39, GH42, GH43, GH51, GH52, GH53, GH63, GH65, GH66, GH67, GH73, GH81, GH84, GH92, GH94, GH95, GH97, GH102, GH103, GH105, GH106, GH108, GH120, GH123, GH125, GH127, GH15, GH141, GH144, GH151, GH161, GH163

## Supplementary Materials

The following are available online. Figure S1. Clustering of metagenomic contigs by MyCC; Figure S2. Estimation of the replication percentages obtained by iRep for cluster 7 of Site A (blue) and clusters 2, 4, and 6 of Site B (green); Table S1. Cluster scores identified by MyCC; Table S2. Cluster validation score by CheckM; Table S3. Stats for the clusters selected for iRep analysis; Table S4. CAZymes annotation in Site A; Table S5. CAZymes annotation in Site B; Table S6. CAZymes annotation in Site C.
