# Supplementary figures and images for: Spatial Metagenomics of Three Geothermal Sites in Pisciarelli Hot Spring Focusing on the Biochemical Resources of the Microbial Consortia
Source: Molecules. 2020 Sep 3;25(17):4023. doi: 10.3390/molecules25174023 (PMC7570011; doi:10.3390/molecules25174023)

Figure S1

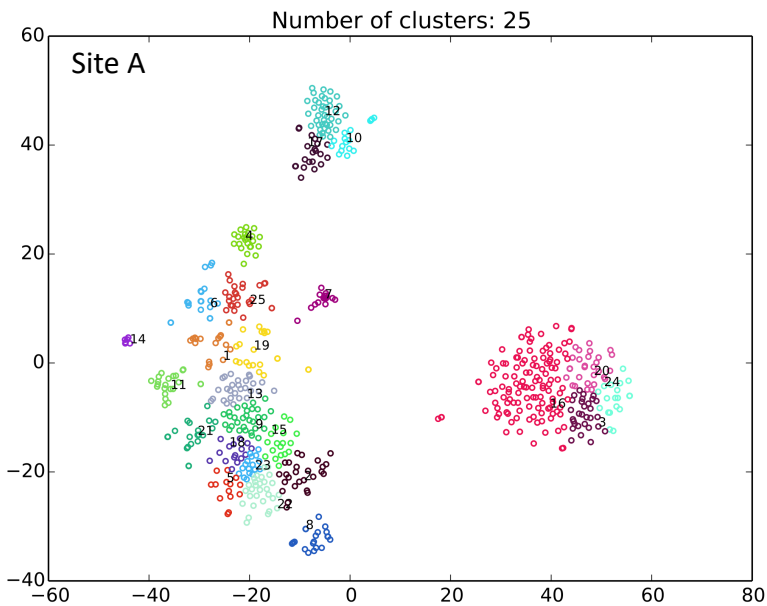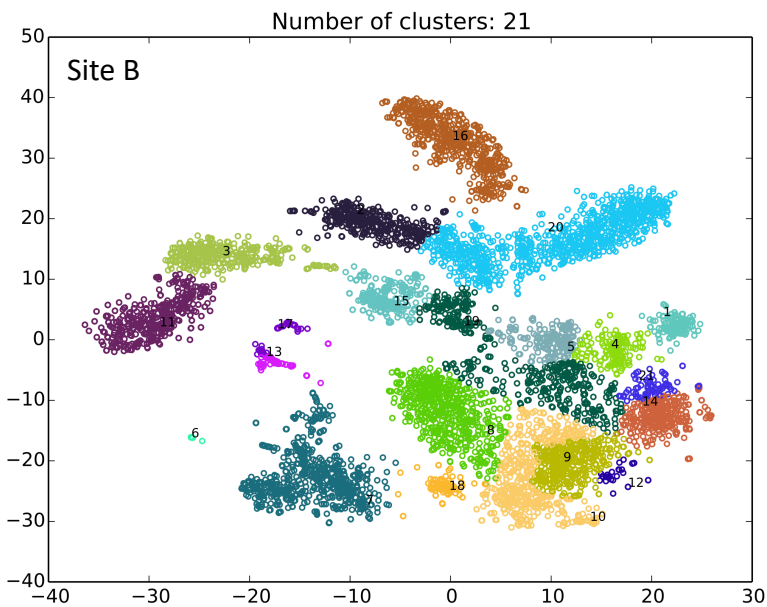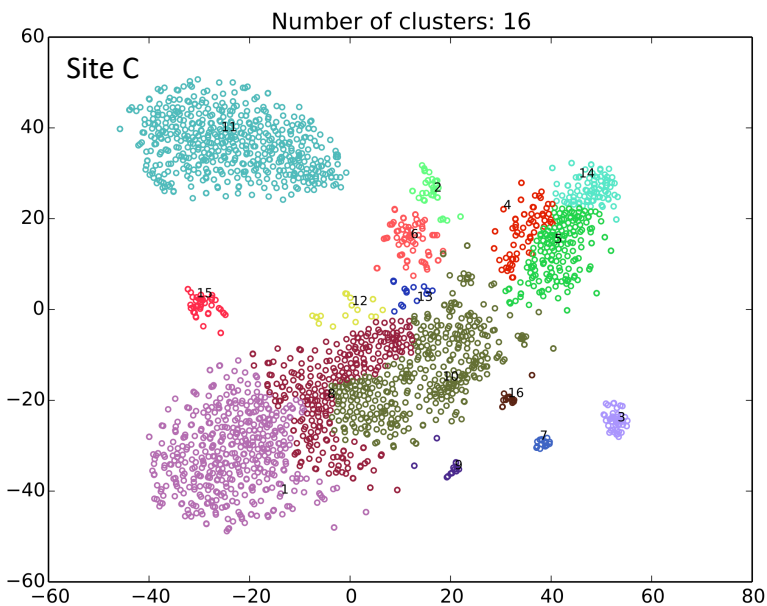

Figure S2

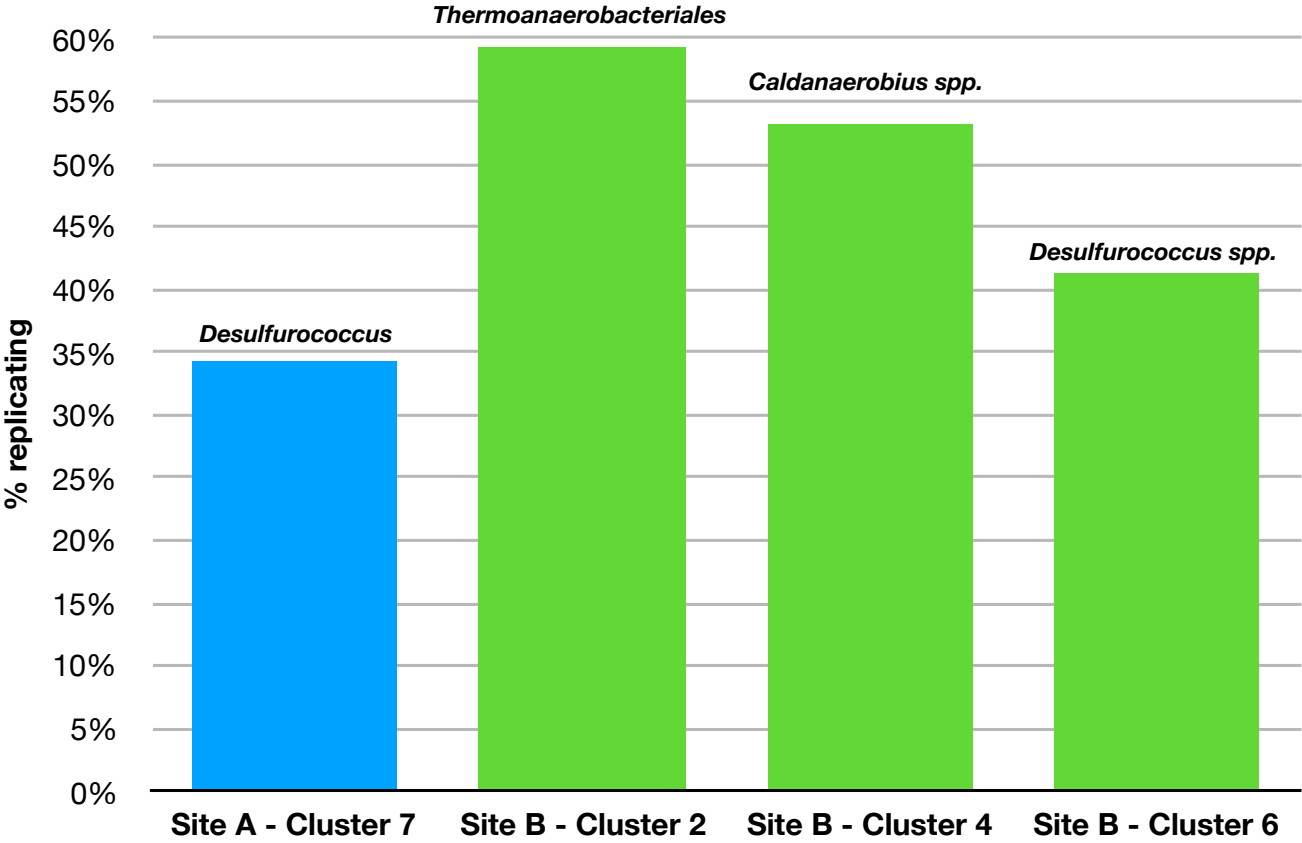

Supplement: Supplementary file 1 [file molecules-25-04023-s001.zip › Supplementary material/Iacono_et_al_Supp_figs.pdf]
